# Association between dietary inflammatory index and all-cause mortality in patients with osteoporosis: data from NHANES

**DOI:** 10.3389/fnut.2025.1579331

**Published:** 2025-05-30

**Authors:** Teng-di Fan, Di-kai Bei, Song-wei Li

**Affiliations:** Department of Orthopedics, Ningbo Medical Center Lihuili Hospital, Ningbo, China

**Keywords:** dietary inflammatory index, all-cause mortality, osteoporosis, cohort study, NHANES

## Abstract

**Background:**

Osteoporosis is a common bone metabolic disease that poses a serious risk of fracture and death. The dietary inflammatory index (DII) is a tool for assessing the impact of diet on the inflammatory response in the body. This study aims to investigate the association of DII with the all-cause mortality of those patients.

**Methods:**

The study population was screened from the National Health and Nutrition Examination Survey (NHANES), after applying the exclusion criteria of age <18 years or missing information on DII, femoral bone marrow density (BMD), age, sex, ethnicity, and other variables. The DII was calculated according to the questionnaire interview of 24-h dietary data. Data analysis methods included the t-test, chi-squared test, weighted Cox regression, Kaplan–Meier (KM) survival analysis, XGBoost analysis, Pearson’s analysis, and interaction analysis.

**Results:**

A total of 361 patients were included in the study, comprising 264 women and 97 men. The Cox regression analysis results indicated that DII, glycohemoglobin, BMI, weight, age, race, and diabetes were independent factors for all-cause mortality (all *p* < 0.05). In addition, patients with higher DII levels had a higher risk of all-cause mortality than those with lower DII (*p* < 0.05). The KM survival curves indicated that patients with lower DII levels had a longer survival time (*p* < 0.05). The variable importance ranking, including DII and other variables, was as follows: age, weight, BMI, glycohemoglobin, DII, ethnicity, and diabetes. Pearson’s correlation analysis further indicated that DII was not significantly correlated with any of them.

**Conclusion:**

Lower DII is independently associated with longer survival time in patients with osteoporosis.

## 1 Introduction

Osteoporosis is a prevalent and debilitating condition characterized by low bone mass and structural deterioration of bone tissue, leading to an increased risk of fractures. It is estimated that over 200 million people worldwide suffer from osteoporosis, and the numbers show an upward trend due to an increase in the aging population and sedentary lifestyle choices (1–3). Osteoporotic hip fractures are particularly devastating, accounting for up to 5% of total mortality (4). However, a significant percentage of individuals, ranging from 21 to 30%, succumb to mortality within a year following such fractures (5). Inflammation plays a pivotal role in bone metabolism, where it can promote bone resorption while inhibiting bone formation (6), consequently leading to osteoporosis (7). The body’s inflammatory state is closely associated with dietary patterns, as proper nutritional status may help mitigate systemic inflammation (8). Studies indicate that minerals, proteins, fruits, and vegetables serve as crucial factors in preventing osteoporosis and fragility fractures (9). Different dietary patterns have varying impacts on skeletal health (10).

The dietary inflammatory index (DII) was first developed by Cavicchia et al. (11) based on the concept that certain dietary factors can trigger an inflammatory response in the body. It is a tool designed to measure the inflammatory potential of an individual’s diet. Later, Shivappa et al. (12) updated the DII. Many studies have shown that higher DII scores are linked to an increased risk of developing cardiovascular disease, diabetes, obesity, and certain types of cancer (13–16). Numerous observational studies have assessed whether a higher DII score is indicative of an escalated risk of death. A notable observational study conducted by Shivappa et al. (17) demonstrated that a higher DII score was associated with increased all-cause mortality as well as mortality related to digestive cancer, cardiovascular disease (CVD), coronary heart disease (CHD), and chronic obstructive pulmonary disease (COPD). Zucchetto et al. (18) revealed that there was no association between the DII and the survival of women with breast cancer via a case–control study. Zucchetto et al. (19) reported that DII was strongly associated with both all-cause and prostate cancer-specific mortality.

However, the relationship between DII and all-cause mortality in osteoporotic patients remains largely explored. Therefore, this study aims to investigate the association between DII and mortality in individuals with osteoporosis, with a specific focus on the role of dietary inflammation in influencing mortality risk.

## 2 Materials and methods

### 2.1 Data source and study population

**Figure 1:**
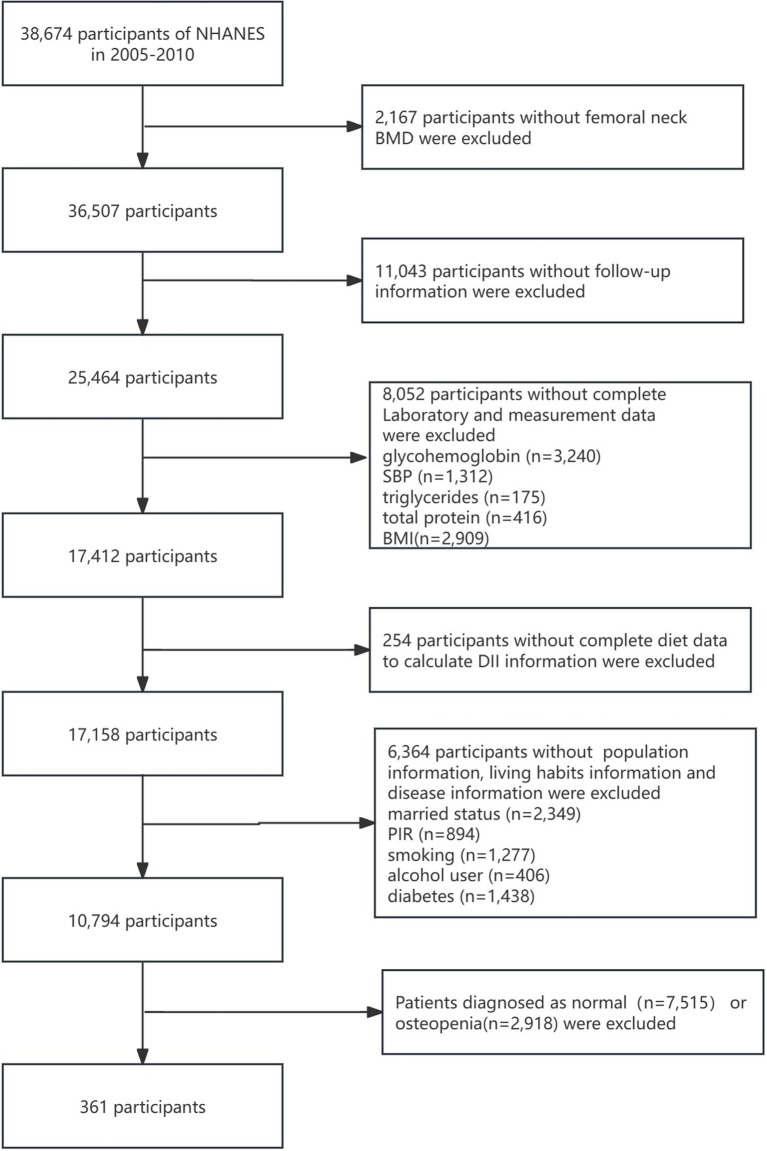
Detailed flowchart of screening of osteoporosis patients. NHANES, National Health and Nutrition Examination Survey; BMD, bone marrow density; SBP, systolic blood pressure; BMI, body mass index; DII, dietary inflammatory index; PIR, poverty–income ratio.

The National Health and Nutrition Examination Survey (NHANES) is a public database based on a nationally representative American population sample (20). Bone mineral density (BMD) was measured from the NHANES 2005–2006 cycle; however, the femoral neck and lumbar spine were not measured in the 2011–2012 cycle. From the 2013–2014 cycle, only the BMD of people aged> 40 years was measured. Therefore, we selected the BMD measurement data at all ages from the 3-year cycles of NHANES: 2005–2006, 2007–2008, and 2009–2010. There were 31,034 people in the three cycles. After a series of exclusions, a total of 361 patients diagnosed with osteoporosis who had complete data on other variables were included, comprising 264 women and 97 men. We calculated the minimum required sample size using G*Power software, assuming an effect size of d = 0.6, an *α* error probability of 0.05, a power (1-*β* error probability) of 0.95, and an allocation ratio (N2/N1) of 1, This calculation yielded a minimum required sample size of 154. Therefore, the 361 enrolled participants meet the analysis requirements. Moreover, to avoid poor stability and overfitting due to the small sample size, we used the “RSM” package in R software to calculate the shrinkage factor. The result indicated a shrinkage factor of 0.982, which falls within an ideal and acceptable range. The study population flowchart is shown in Figure 1. Notably, the 361 osteoporosis patients included in the analysis represent an estimated 4,489,709 osteoporosis patients in the US, based on weighted calculation.

### 2.2 Diagnosis of osteoporosis and mortality outcome

According to the World Health Organization (WHO), osteoporosis is defined as a *T*-score of −2.5 or less of the femoral neck. Osteopenia is defined as a *T*-score of more than one but less than 2.5. T scores were calculated based on the following equation using BMD of the femoral neck: T = BMD (target)- mean BMD (reference group)/standard deviation (reference group). The reference group comprised participants who were female, white, and aged 20–29 years according to the WHO International Reference (21).

Mortality status and follow-up time were acquired from the National Death Index (NDI) database up to 31 December 2019. The NDI is the death information record database of the corresponding participants in the NHANES, which records the survival time and survival status of the participants and is paired with the samples in the NHANES database.^1^ All-cause mortality was defined as the primary outcome of our study.

### 2.3 Definition of DII

The DII is a tool for assessing the potential pro-inflammatory and anti-inflammatory effects of an individual’s dietary intake. It is based on a systematic review of the scientific literature on dietary components and their effects on inflammatory markers such as IL-1β, IL-4, IL-6, IL-10, TNF-*α*, and C-reactive protein (CRP). Each study involving a food parameter is assigned one of the three possible effects: pro-inflammatory (+1), anti-inflammatory (−1), or no effect (0). To enhance the precision of the DII, the type of literature and study design are weighted accordingly when calculating each food’s inflammatory score. The DII score ranges from +7.98, indicating a maximally pro-inflammatory diet, to −8.87, indicating a maximally anti-inflammatory diet. The calculation of an individual’s DII involves several steps: (1) The reported daily intake of each dietary component or nutrient is first standardized by subtracting the global average daily intake (i.e., the reference standard value) and dividing it by the standard deviation of that global average. (2) This value, known as a Z-score, represents the individual’s exposure to that food component relative to a “global average.” The Z-scores are then converted to percentiles. (3) The percentile for each dietary component is multiplied by its overall inflammatory effect score. (4) Finally, the individual scores for all dietary components are summed to derive the overall DII score. A higher DII score indicates a stronger pro-inflammatory effect, while a lower DII score suggests a stronger anti-inflammatory effect. Thus, the DII provides a quantitative estimate of the inflammatory potential of an individual’s overall diet (12).

The 24-h dietary data of the NHANES were collected, including 28 nutritional ingredients: energy, protein, carbohydrate, fiber, total fat, saturated fat, monounsaturated fatty acids, polyunsaturated fatty acids, cholesterol, vitamin A, *β*-carotene, thiamin, riboflavin, niacin, Vitamin B6, folic acid, vitamin B12, vitamin C, vitamin D, vitamin E, magnesium, iron, zinc, selenium, caffeine, alcohol, and N3 and N6 fatty acids (22). The DII was calculated via reported methods (12) based on the dietary data. Supplementary File 1 shows the calculation process of DII in detail.

### 2.4 Covariates

The variables included: age; sex (female and male); ethnicity (Mexican American, Non-Hispanic Black, Non-Hispanic White, other Hispanic, and other ethnicities); marital status (never married, living with partner, married, divorced, separated, and widowed); education level (less than 9th grade, 9–11th grade, high school graduate/GED or equivalent, some college or AA degree, and college graduate or above); glycohemoglobin (%); systolic blood pressure (SBP, mmHg); diastolic blood pressure (DBP, mmHg); triglycerides (mg/dL); uric acid (mg/dL); total calcium (mmol/L); total cholesterol (mg/dL); total protein, (g/dL); height (cm); body mass index (BMI, kg/m^2^); poverty–income ratio (PIR, %); weight (kg); smoking status (never, former, current); alcohol use (never, former, mild, moderate, heavy); hypertension (yes or no); diabetes (no, impaired glucose tolerance, yes); and history of fracture (yes or no).

An alcohol user is classified as follows: (1) a participant who had less than 12 drinks in a lifetime was identified as a never alcohol user; (2) a participant who had equal to or more than 12 drinks in 1 year and did not drink last year, or did not drink in the last year but drank equal to or more than 12 drinks in a lifetime, was identified as a former alcohol user; (3) a female (or male) participant who has equal to or less than 1 (or 2) drinks daily on average during the last year was identified as a mild drinker; (4) A female (or male) participant who has equal to or less than 2 (or 3) drinks daily on average during the last year was identified as a moderate drinker; and (5) a female (or male) participant who has equal to or more than 3(or 4) drinks daily on average during the last year was identified as a heavy drinker (23, 24). A smoker is classified as follows: (1) current smokers were defined as adults who smoked more than 100 cigarettes in life and smoked some days or every day; (2) former smokers were defined as adults who do not currently smoke cigarettes; and (3) never smokers were defined as adults who smoked less than 100 cigarettes in their lifetime.

BMI was calculated as follows equation: BMI = weight (kg) /height squared (m^2^) (25).

Hypertension was diagnosed according to any of the three conditions (1) the participant’s answer to the questionnaire, for example, “BPQ020 - Ever told you had high blood pressure,” was yes; (2) the participant had SBP measurement equal to or greater than 140 mmHg and/or DBP measurement equal to or greater than 90 mmHg; and (3) the participant reported current take antihypertensive medication (26). Diabetes was identified as present if any of the following four criteria were met: (1) the participant’s answer to the questionnaire, for example, “DIQ010 - Doctor told you have diabetes,” was yes; (2) glycohemoglobin levels were higher than 6.5%; (3) randomly assigned blood glucose levels were equal to or greater than 11.1 mmol/L; and (4) the participant was taking medications for diabetes or insulin (22). In addition, the 2-h oral glucose tolerance test blood glucose (mmol/L) in the range of 7.8–11.0 mmol/L is considered impaired glucose tolerance among participants who do not meet the criteria for diabetes diagnosis. Fractures were defined based on responses to the following three questions: “OSQ010a - Broken or fractured a hip”; “OSQ010b - Broken or fractured a wrist”; and “OSQ010c - Broken or fractured spine.” A fracture was identified if any of the three questions received a “Yes” response.

### 2.5 Statistical analysis

All samples were divided into two groups according to survival status (alive vs. deceased). Categorical variables were expressed as frequencies and percentages. Continuous variables were expressed as mean ± standard deviation. Subsequently, the difference between the two groups was compared using the t-test for continuous variables, while the chi-squared test was used to compare the categorical variables.

The dietary day one sample weight (WTDRD1/3) was used for weighted analyses.^2^ The univariate Cox regression was used to explore the association between all variables and all-cause mortality. We then explored the association between the DII (DII quartiles) and all-cause mortality using three Cox regression models: crude model (no adjustment), model 1 (adjusted for age, sex, and ethnicity), and model 2 (further adjusted for glycohemoglobin, systolic and diastolic blood pressure, uric acid, BMI, PIR weight, hypertension, and diabetes).

The Kaplan–Meier (KM) analysis was then performed to reveal the survival difference of patients with osteoporosis between different DII level groups using the log-rank test. All the patients were divided into high- and low-level groups based on the DII cutoff value determined using the “maxstat” package in R software. The survival difference between the high- and low-level groups was then compared. Additionally, survival differences among the four DII quartiles were investigated. To explore the contribution of each independent factor to all-cause mortality, variable importance rankings were acquired using the Python XGBoost 1.2.1 method. Pearson’s analysis was performed to explore the correlation between DII and other independent impact factors. Subgroup analysis was used to explore the relationship between DII and all-cause mortality in subgroups. Interaction analysis was used to explore the interaction between DII and subgroup variables on all-cause mortality. IBM SPSS 23.0 software and R software 4.2.2 were used to analyze data, and a *p*-value of <0.05 was considered statistically significant.

## 3 Results

### 3.1 Baseline characteristics

The baseline characteristics are shown in Table 1. There were significant differences between the deceased group and the alive group in terms of glycohemoglobin, DII, SBP, DBP, uric acid, BMI, PIR, weight, age, survival time, ethnicity, marital status, hypertension, and diabetes (all *p* < 0.05). Among them, DII (2.059 vs. 1.519), age (72.957 vs. 59.150), glycohemoglobin (6.024 vs. 5.671), SBP (139.090 vs. 128.122), and uric acid (5.379 vs. 5.004) in the deceased group were significantly higher than those in the alive group. The hypertension rate and diabetes rate in the deceased group were 25.015 and 21.279% higher than those in the alive group, respectively.

**Table 1:** Clinical characteristics of patients with osteoporosis.

Characteristics	Alive (*n* = 205)	Deceased (*n* = 156)	*p*
Glycohemoglobin, (%)	5.671 (0.059)	6.024 (0.150)	0.023
DII	1.519 (0.210)	2.059 (0.131)	0.019
SBP, (mmHg)	128.122 (1.601)	139.090 (3.174)	0.005
DBP, (mmHg)	68.779 (1.343)	62.362 (1.753)	0.004
Triglycerides, (mg/dL)	153.369 (10.788)	160.043 (6.994)	0.601
Uric acid, (mg/dL)	5.004 (0.083)	5.379 (0.154)	0.044
Total calcium, (mmol/L)	2.382 (0.011)	2.355 (0.014)	0.091
Total cholesterol, (mg/dL)	211.105 (2.914)	200.213 (5.297)	0.094
Total protein, (g/dL)	7.040 (0.031)	6.994 (0.052)	0.407
Height, (cm)	162.373 (0.912)	160.558 (0.824)	0.143
BMI, (kg/m^2^)	25.748 (0.500)	23.991 (0.638)	0.029
PIR, (%)	2.821 (0.161)	2.301 (0.129)	0.017
Weight, (kg)	68.079 (1.713)	62.283 (2.188)	0.029
Age, (years)	59.150 (1.151)	72.957 (0.784)	< 0.001
Survival time, (month)	149.938 (2.731)	75.565 (3.765)	< 0.001
Sex, *n* (%)			0.637
Female	149 (72.775)	115 (75.432)	
Male	56 (27.225)	41 (24.568)	
Race, *n* (%)			0.027
Mexican American	47 (9.372)	13 (2.637)	
Non-Hispanic Black	14 (3.088)	15 (4.459)	
Non-Hispanic White	114 (75.516)	117 (87.152)	
Other Hispanic	17 (4.381)	5 (1.012)	
Other Race - Including Multi-Racial	13 (7.643)	6 (4.740)	
Marital status, *n* (%)			<0.001
Never married	20 (8.478)	6 (3.944)	
Living with partner	6 (3.416)	1 (0.566)	
Married	117 (58.497)	54 (36.928)	
Divorced	24 (14.730)	21 (14.079)	
Separated	4 (0.803)	2 (0.998)	
Widowed	34 (14.075)	72 (43.485)	
Education level, *n* (%)			0.148
Less Than 9th Grade	37 (9.350)	29 (14.236)	
9-11th Grade	31 (12.568)	34 (21.792)	
High School Grad/GED or Equivalent	59 (29.904)	42 (26.127)	
Some College or AA degree	51 (32.416)	32 (24.150)	
College Graduate or above	27 (15.762)	19 (13.695)	
Smoker, *n* (%)			0.458
Never	117 (50.689)	70 (46.278)	
Former	46 (24.157)	53 (30.685)	
Now	42 (25.154)	33 (23.038)	
Alcohol user, *n* (%)			0.101
Never	61 (25.354)	44 (27.383)	
Former	36 (19.134)	54 (32.802)	
Mild	54 (29.657)	38 (25.274)	
Moderate	26 (13.461)	10 (7.207)	
Heavy	28 (12.395)	10 (7.334)	
Hypertension, *n* (%)			<0.001
No	101 (54.916)	52 (29.901)	
Yes	104 (45.084)	104 (70.099)	
Diabetes, *n* (%)			<0.001
No	148 (79.885)	92 (59.866)	
Impaired glucose tolerance	26 (9.611)	15 (8.351)	
Yes	31 (10.504)	49 (31.783)	
Fracture			0.240
No	159 (78.422)	112 (71.584)	
Yes	46 (21.578)	44 (28.416)	

Continuous variables were reported as mean ± standard deviation. *p* values were obtained from independent t-tests. Categorical variables were expressed as frequencies and percentages. *p* values were obtained from chi-square tests. DII, dietary inflammatory index; SBP, systolic blood pressure; DBP, diastolic blood pressure; BMI, body mass index; PIR, poverty-income ratio.

### 3.2 Cox regression analysis

The results of the univariate Cox analysis are shown in Table 2. DII, glycohemoglobin, SBP, DBP, uric acid, BMI, PIR, weight, age, ethnicity, hypertension, and diabetes were related to all-cause mortality (all *p* < 0.05). To confirm the significant association between DII and all-cause mortality, three models were established. Patients in the Q4 group had a higher risk of all-cause mortality than the Q1 group (crude model: HR 95%CI: 2.483(1.507, 4.091); Model 1 HR95%CI: 2.522(1.469, 4.331); Model 2: 2.121(1.241, 3.625); all *p* < 0.05, Table 3). The risk of all-cause mortality increased with the increase of DII (P for trend < 0.001, Table 3). In addition, the independent roles of glycohemoglobin, BMI, weight, age, race, and diabetes were also observed in Model 2 (all *p* < 0.05, Supplementary Table 1).

**Table 2:** Univariate Cox analysis between all-cause mortality and DII in patients with osteoporosis.

Characteristics	HR (95%CI)	*p*
Glycohemoglobin	1.203 (1.077, 1.344)	0.001
DII		
Q1	ref	ref
Q2	1.554 (0.905, 2.669)	0.110
Q3	1.614 (0.961, 2.711)	0.070
Q4	2.483 (1.507, 4.091)	<0.001
SBP	1.014 (1.008, 1.019)	<0.001
DBP	0.985 (0.978, 0.993)	<0.001
Triglycerides	1.000 (0.998, 1.002)	0.910
Uric acid	1.163 (1.048, 1.292)	0.005
Total calcium	0.087 (0.008, 0.904)	0.041
Total cholesterol	0.994 (0.988, 1.000)	0.058
Total protein	0.779 (0.477, 1.272)	0.318
Height	0.981 (0.959, 1.003)	0.087
BMI	0.938 (0.894, 0.985)	0.010
PIR	0.821 (0.713, 0.946)	0.006
Weight	0.977 (0.959, 0.997)	0.021
Age	1.080 (1.061, 1.098)	<0.001
Sex		
Female	ref	ref
Male	0.832 (0.533, 1.300)	0.420
Race		
Non-Hispanic White	ref	ref
Non-Hispanic Black	1.330 (0.639, 2.766)	0.446
Mexican American	0.287 (0.140, 0.590)	<0.001
Other Hispanic	0.257 (0.074, 0.899)	0.033
Other Race	0.625 (0.235, 1.662)	0.346
Marital status		
Living with partner	ref	ref
Never Married	2.715 (0.325, 22.662)	0.356
Married	3.817 (0.463, 31.493)	0.213
Widowed	12.264 (1.538, 7.790)	0.018
Divorced	5.639 (0.699, 45.495)	0.104
Separated	8.633 (0.388, 192.208)	0.173
Education level		
Less Than 9th Grade	ref	ref
9-11th Grade	1.040 (0.599, 1.805)	0.890
High School Grad/GED or Equivalent	0.693 (0.407, 1.181)	0.178
Some College or AA degree	0.626 (0.325, 1.202)	0.159
College Graduate or above	0.616 (0.306, 1.241)	0.175
Smoke		
Never	ref	ref
Former	1.267 (0.794, 2.021)	0.320
Now	0.957 (0.653, 1.402)	0.821
Alcohol user		
Never	ref	ref
Former	1.357 (0.859, 2.145)	0.191
Mild	0.792 (0.382, 1.643)	0.531
Moderate	0.535 (0.203, 1.409)	0.206
Heavy	0.560 (0.280, 1.118)	0.100
Hypertension		
No	ref	ref
Yes	2.319 (1.530, 3.515)	<0.001
Diabetes		
No	ref	ref
Yes	2.656 (1.695, 4.161)	<0.001
Fracture		
No	ref	ref
Yes	1.360 (0.885, 2.090)	0.160

Univariate Cox regression analysis calculated the HR (95%CI). DII, dietary inflammatory index; SBP, systolic blood pressure; DBP, diastolic blood pressure; BMI, body mass index; PIR, poverty-income ratio; HR, hazard ratio; CI, confidence interval.

**Table 3:** Cox regression analysis on the association between DII and all-cause mortality in osteoporosis patients.

DII (quartile value, *n*)	Crude model		Model 1		Model 2	
	HR (95%CI)	*p*	HR (95%CI)	*p*	HR (95%CI)	*p*
Q1[(−3.463, 0.780), *n* = 91]	ref		ref		ref	
Q2[(0.789, 2.146), *n* = 90]	1.554 (0.905, 2.669)	0.110	1.487 (0.760, 2.911)	0.247	1.331 (0.711, 2.491)	0.371
Q3[(2.202, 3.264), *n* = 90]	1.614 (0.961, 2.711)	0.070	1.577 (0.983, 2.532)	0.059	1.351 (0.839,2.175)	0.216
Q4[(3.271, 4.727), *n* = 90]	2.483 (1.507, 4.091)	<0.001	2.522 (1.469, 4.331)	<0.001	2.121 (1.241, 3.625)	0.006
P for trend		<0.001		0.002		0.017

Multivariate Cox regression analysis calculated the HR (95%CI) in different models. Crude model: without adjustment. Model 1: adjusted for age, sex, and race. Model 2: further adjusted for glycohemoglobin, systolic and diastolic blood pressure, uric acid, BMI, poverty-income ratio, weight, hypertension, and diabetes. DII, dietary inflammatory index; HR, hazard ratio; CI, confidence interval; BMI, body mass index; PIR, poverty-income ratio.

### 3.3 KM analysis

**Figure 2:**
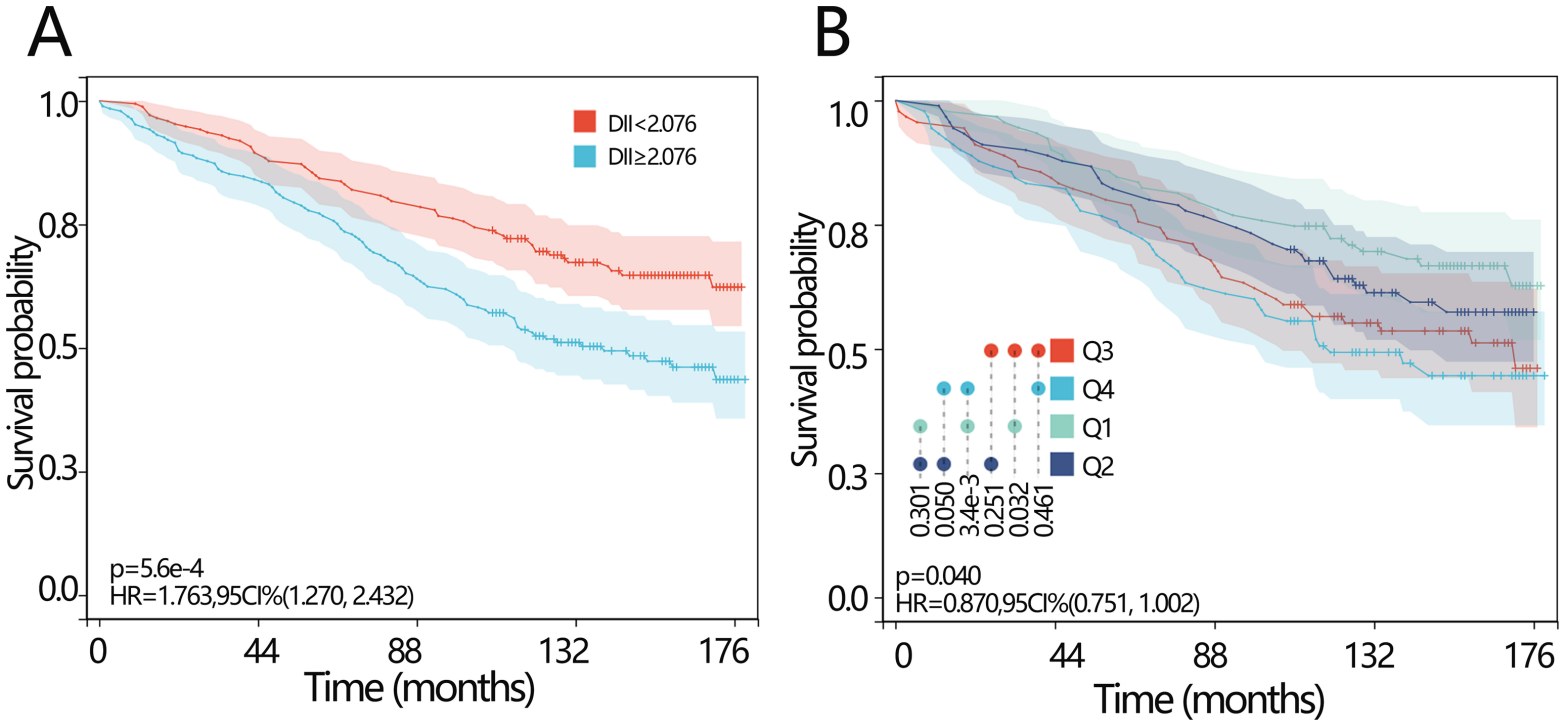
Prognosis values of DII in osteoporosis patients. **(A)** The KM survival curve of all-cause mortality based on the DII for the patients with osteoporosis according to curve according to the optimal cutoff score of the DII. **(B)** The KM survival curve of all-cause mortality based on the DII for the patients with osteoporosis according to the curve according to the quartile of the DII. DII, dietary inflammatory index; KM, Kaplan–Meier; HR, hazard ratio; CI, confidence interval.

The KM survival curves were then plotted to investigate the survival difference between different DII-level groups. Patients with lower DII levels (<2.076) had a longer survival time (Figure 2A, HR = 1.763, *p* < 0.001). When the DII was set as a categorical variable according to its quartile, the median survival time in the Q4 group was significantly lower than that in the Q1 group (Figure 2B, *p* = 0.003).

### 3.4 Correlation analysis

**Figure 3:**
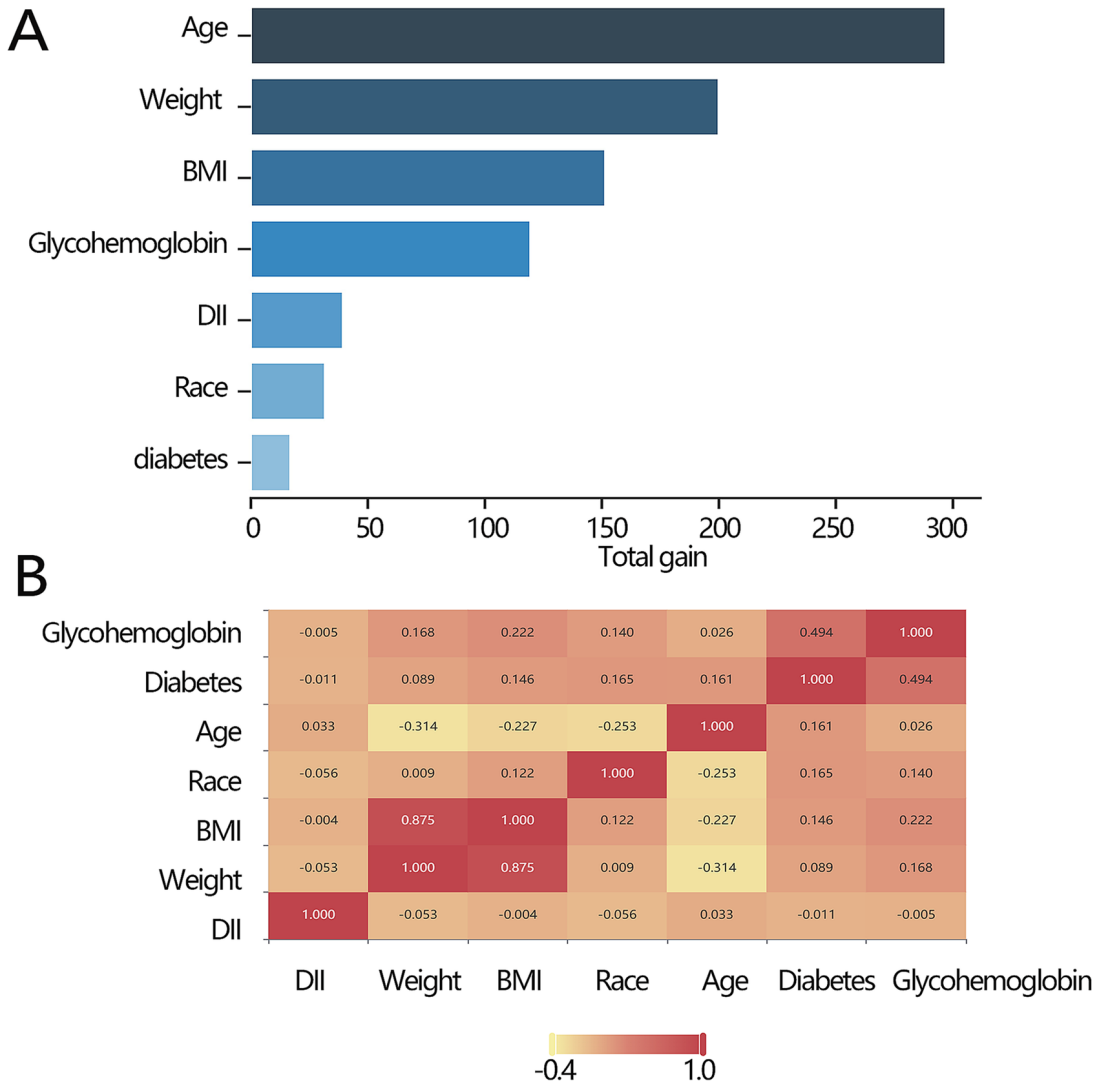
Association between the key factors and all-cause mortality. **(A)** The importance ranking bar chart of key factors. **(B)** The heatmap of the key factors. DII, dietary inflammatory index; BMI, body mass index.

The above results have demonstrated the importance of DII in all-cause mortality. We further explored the importance of DII among several independent variables. The importance ranking was as follows: age>weight>BMI > glycohemoglobin>DII > ethnicity>diabetes (Figure 3A). Pearson’s correlation analysis was used to explore the correlation between DII and other independent impact factors, and the results demonstrated that there was no significant relationship between DII and any of them (all *p* > 0.05, Figure 3B).

### 3.5 Subgroup analysis

We conducted subgroup analyses to investigate the sensitivity of the relationship between DII and all-cause mortality. The results showed that DII was significantly associated with all-cause mortality in the following groups: patients with fractures, patients aged>70 years, women, patients with a weight range of 33.2–61.5 kg, and patients with hypertension (all *p* < 0.05, Table 4). Moreover, the interaction results showed that DII had no interaction with these variables on all-cause mortality (all *p* > 0.05, Table 4).

**Table 4:** Cox analysis between all-cause mortality and DII in subgroups.

Variable	HR [95% CI]	*p*	P for interaction
Age			0.835
[23, 70] (*n* = 188, deceased = 40)	1.131 (0.898, 1.424)	0.295	
[70, 85] (*n* = 173, deceased = 116)	1.109 (1.003, 1.227)	0.044	
Sex			0.834
Female (*n* = 264, deceased = 115)	1.176 (1.039, 1.332)	0.010	
Male (*n* = 97, deceased = 41)	1.133 (0.854, 1.503)	0.386	
Fracture			0.182
No (*n* = 271, deceased = 112)	1.111 (0.958, 1.288)	0.165	
Yes (*n* = 90, deceased = 44)	1.344 (1.084, 1.668)	0.007	
Weight			0.096
[33.2, 61.5] (*n* = 183, deceased = 95)	1.283 (1.084, 1.519)	0.004	
[61.5, 120.9] (*n* = 178, deceased = 61)	1.051 (0.897, 1.232)	0.536	
Hypertension			0.394
No (*n* = 154, deceased = 52)	1.270 (0.980, 1.645)	0.071	
Yes (*n* = 207, deceased = 104)	1.124 (1.012, 1.249)	0.030	

Univariate Cox regression analysis calculated the HR (95%CI). The population was divided into two groups according to the median age (or the median weight). DII, dietary inflammatory index; HR, hazard ratio; CI, confidence interval.

## 4 Discussion

This study assesses the association between the mortality of patients with osteoporosis and DII based on the NHANES 2005–2010. We found that DII was independently and positively associated with the all-cause mortality of patients with osteoporosis. Moreover, osteoporosis patients with lower DII levels had a longer survival time. These findings indicated that a low inflammatory diet pattern may be necessary to prevent mortality in patients with osteoporosis.

DII is associated with inflammatory cytokines such as C-reactive protein, IL-6, and homocysteine. It is calculated based on whether a particular food significantly increases levels of IL-1β, IL-6, TNF-*α*, and C-reactive protein or reduces levels of IL-4 and IL-10 (12, 27–29). A high DII is associated with high levels of inflammatory cytokines such as TNF-α, IL-1, IL-6, IL-7, and PGE2. These inflammatory cytokines can impact the biological pathway of bone metabolism by inducing the expression of M-CSF and RANKL, stimulating osteoclast-mediated bone resorption, and reducing bone mineral density (30, 31), thereby increasing the risk of fractures (32–34). In addition, a high-DII diet can lead to high levels of inflammation and oxidative stress in the body, contributing to the development of risk factors that accelerate and aggravate biological aging (35). These risk factors can cause cell dysfunction, tissue degradation, and organ damage, thereby accelerating the aging process (36). An increase in senescent cells and the senescence-related secretory phenotype led to elevated osteocyte apoptosis and the imbalance of the bone metabolism pathway, resulting in reduced bone mass and the development of osteoporosis. Bone metabolism pathway includes the Wnt pathway (LRP5, SOST, WNT10B, WNT16, SFRP1, FOXC2, LRP4, GPR177, and CTNNB1), the RANK pathway (RANKL, RANK, and OPG), the vitamin D pathway (VDR and DBP), estrogen signaling pathway (ESR1, ESR2, and CYP19A1) (37).

In the overall analysis of patients with osteoporosis, no significant difference in fracture incidence was observed between the deceased and alive groups. This lack of difference may be attributed to population-wide heterogeneity, such as variation in fracture types, severity, and comorbid conditions, which can obscure specific risk patterns in certain subgroups. However, subgroup analysis among patients with fractures revealed that a higher DII was significantly associated with increased mortality risk. This finding suggests that diet-related chronic inflammatory states may exacerbate systemic inflammatory responses post-fracture, thereby elevating the risk of complications such as infection, thrombosis, or impaired fracture healing. For instance, the release of pro-inflammatory cytokines may contribute to muscle catabolism, vascular endothelial injury, and metabolic dysregulation, thereby collectively influencing survival outcomes (12, 38). Moreover, elevated DII may indirectly worsen systemic pathophysiological disturbances in osteoporotic patients by affecting bone metabolism—specifically by inhibiting osteoblast differentiation and enhancing osteoclast activity—thereby further reducing life expectancy (39). We also observed that patients in the deceased group with osteoporosis were older than those in the alive group, and the relationship between DII and all-cause mortality was more significant in the older age group. These findings are consistent with previous research conclusions. In elderly individuals, osteoporosis may lead to other diseases due to being bedridden, including excessive blood loss as well as cardiovascular and respiratory diseases, thus potentially resulting in death (3, 40). These results suggest that special attention should be paid to osteoporosis patients with fractures and the elderly.

The significant correlation observed between the DII and osteoporosis prognosis in this study suggests that anti-inflammatory dietary interventions (such as increasing the intake of *ω*-3 fatty acids, polyphenols, and dietary fiber, and controlling the proportion of pro-inflammatory foods) could be integrated into comprehensive osteoporosis treatment regimens in clinical practice. It is also recommended that the dynamic monitoring of inflammatory markers (for example, CRP and IL-6) be incorporated into an optimized treatment outcome assessment system (41). At the public health level, it is suggested that dietary inflammation assessment be included in screening criteria for high-risk osteoporosis populations and that simple DII self-assessment tools should be developed to assist in community health management (42). Additionally, specific subsidy policies should be implemented to increase the availability of anti-inflammatory foods, which may have potential value in reducing the incidence of osteoporotic fractures. Policymakers should consider incorporating nutritional interventions into osteoporosis prevention and treatment guidelines and improving food labeling systems based on the “food inflammatory load” labeling framework (43). This finding mechanistically supports the core hypothesis that chronic inflammation exacerbates bone resorption through the activation of the RANKL/NF-κB pathway (44, 45). Future multicenter intervention trials are needed to validate the actual effects of dietary modulation on bone mineral density improvement in osteoporosis patients and to explore personalized nutritional strategies based on genetic polymorphisms, aiming to reduce fracture risk and mortality in this patient population.

## 5 Strengths and limitations

Our results are consistent with those of Ke et al. (46). Compared to their study, our research methods are more systematic and rigorous, incorporating KM curves, mediation analysis, and XGBoost analysis. Additionally, we considered fractures—an important cause of death in osteoporosis patients—that enhance the reliability of our conclusions. However, our study did not consider the impact of certain comorbid diseases, such as chronic kidney stones and cardiovascular disease. In addition, the difference between the two studies is also reflected in the study population. Ke et al. included participants with osteopenia and osteoporosis, while ours only included those with osteoporosis. This difference in inclusion will lead to differences in some research results. For example, our study found that there was no difference in the education levels between the survival group and the deceased group, but their results showed that there was a significant difference in the education levels between the two groups. However, the main conclusions are consistent, revealing that DII is a risk factor for all-cause mortality in patients with osteoporosis. This study has several limitations. First, dietary information was obtained through self-reported questionnaires, which may lead to memory bias due to recall history. Second, follow-up data were only available up to 31 December 2019 and have not been revised since. Third, there is potential racial bias, as the study population primarily consists of individuals from the US. Fourth, the diagnosis of osteoporosis was based solely on femoral neck BMD, which may not completely capture the condition in all patients Fifth, this study did not account for energy outliers. Sixth, the small sample size may introduce bias and reduce the reliability of the conclusions. A larger number of samples is needed to verify the conclusion. Despite these limitations, this study provides a theoretical basis for understanding the association of DII and all-cause mortality in patients with osteoporosis and offers valuable insights for clinical management. Longitudinal studies in an independent cohort will be conducted to further validate our results.

## 6 Conclusion

In this study, we enrolled patients with osteoporosis from the NHANES to explore the association between the DII and all-cause mortality. We found that DII was an independent risk factor for mortality in patients with osteoporosis, and lower DII scores had longer survival times than those with higher DII.

## Data Availability

The raw data supporting the conclusions of this article will be made available by the authors, without undue reservation.

## Footnotes

^1^ https://www.cdc.gov/nchs/data-linkage/mortality.htm

^2^ https://wwwn.cdc.gov/nchs/nhanes/tutorials/default.aspx
