# Supplementary material for: Association between dietary inflammatory index and all-cause mortality in patients with osteoporosis: data from NHANES
Source: Front Nutr. 2025 May 30;12:1579331. doi: 10.3389/fnut.2025.1579331 (PMC12162689; doi:10.3389/fnut.2025.1579331)
Supplement: Supplementary file 1 [file Table_1.docx]

**Supplementary Table 1** Specific results for adjustment variables in Model 2

| Characteristics | HR (95%CI) | P |
| --- | --- | --- |
| DII |  |  |
| Q1 | ref | ref |
| Q2 | 1.331(0.711,2.491) | 0.371 |
| Q3 | 1.351(0.839,2.175) | 0.216 |
| Q4 | 2.121(1.241,3.625) | 0.006 |
| Glycohemoglobin | 1.245(1.045,1.483) | 0.014 |
| SBP | 1.002(0.996,1.009) | 0.476 |
| DBP | 0.999(0.989,1.009) | 0.786 |
| Uric acid | 1.121(0.995,1.263) | 0.061 |
| BMI | 0.823(0.758,0.894) | <0.001 |
| PIR | 0.881(0.750,1.034) | 0.121 |
| Weight | 1.041(1.014,1.069) | 0.003 |
| Age | 1.075(1.055,1.094) | <0.001 |
| Sex |  |  |
| Female | ref | ref |
| Male | 1.724(0.962,3.088) | 0.067 |
| Race |  |  |
| Non-Hispanic White | ref | ref |
| Non-Hispanic Black | 0.991(0.520,1.888) | 0.978 |
| Mexican American | 0.464(0.199,1.086) | 0.077 |
| Other Hispanic | 0.262(0.090,0.757) | 0.013 |
| Other Race | 0.643(0.354,1.169) | 0.148 |
| Hypertension |  |  |
| No | ref | ref |
| Yes | 1.111(0.811,1.522) | 0.513 |
| Diabetes |  |  |
| No | ref | ref |
| Yes | 2.337(1.386,3.938) | 0.001 |

Note: Multivariate Cox regression analysis calculated the HR (95%CI). Abbreviation: DII: dietary inflammatory index, SBP: systolic blood pressure, DBP: diastolic blood pressure, BMI: body mass index, PIR: poverty-income ratio, HR: hazard ratio, CI: confidence interval.
