# Supplementary material for: Association between dietary inflammatory index and all-cause mortality in patients with osteoporosis: data from NHANES
Source: Front Nutr. 2025 May 30;12:1579331. doi: 10.3389/fnut.2025.1579331 (PMC12162689; doi:10.3389/fnut.2025.1579331)
Supplement: Supplementary file 2 [file Table_2.docx]

DII Calculation Supplementary Files

The steps for calculating DII are as follows:

(1) Select 28 Dietary Parameters

The DII calculation involves 28 nutrients or food components identified in the NHANES dataset, including macronutrients, vitamins, minerals, and bioactive compounds (Supplementary Table 1).

(2) Calculate Z-Score

Standardize individual intake against global reference values:

𝑍-score = (Individual daily intake − Global mean intake)/Global standard deviation

(3) Convert to Centered Percentile

Percentile transformation: Convert Z-scores to a percentile value.

Centralization: Centered score = (Percentile × 2) – 1

(4) Calculate DII

DII=∑(Centered score*Inflammatory effect score)

| Table DII component information | | |  | |
| --- | --- | --- | --- | --- |
| Food parameter | Inflammatory effect score | Global daily mean intake (units/d) | | Global standard deviation |
| Alcohol (g) | −0.278 | 13.98 | | 3.72 |
| Vitamin B12 (μg) | 0.106 | 5.15 | | 2.70 |
| Vitamin B6 (mg) | −0.365 | 1.47 | | 0.74 |
| β-Carotene (μg) | −0.584 | 3718.00 | | 1720.00 |
| Caffeine (g) | −0.110 | 8.05 | | 6.67 |
| Carbohydrate (g) | 0.097 | 272.20 | | 40.00 |
| Cholesterol (mg) | 0.110 | 279.40 | | 51.20 |
| Energy (kcal) | 0.180 | 2056 | | 338.00 |
| Total fat (g) | 0.298 | 71.40 | | 19.40 |
| Fibre (g) | −0.663 | 18.80 | | 4.90 |
| Folic acid (μg) | −0.190 | 273.00 | | 70.70 |
| Fe (mg) | 0.032 | 13.35 | | 3.71 |
| Mg (mg) | −0.484 | 310.10 | | 139.40 |
| Monounsaturated Fat (g) | −0.009 | 27.00 | | 6.10 |
| Niacin (mg) | −0.246 | 25.90 | | 11.77 |
| n-3 Fatty acids (g) | −0.436 | 1.06 | | 1.06 |
| n-6 Fatty acids (g) | −0.159 | 10.80 | | 7.50 |
| Protein (g) | 0.021 | 79.40 | | 13.90 |
| Polyunsaturated Fat (g) | −0.337 | 13.88 | | 3.76 |
| Riboflavin (mg) | −0.068 | 1.70 | | 0.79 |
| Saturated fat (g) | 0.373 | 28.60 | | 8.00 |
| Se (μg) | −0.191 | 67.0 | | 25.10 |
| Thiamin (mg) | −0.098 | 1.70 | | 0.66 |
| Vitamin A (RE) | −0.401 | 983.90 | | 518.6 |
| Vitamin C (mg) | −0.424 | 118.20 | | 43.46 |
| Vitamin D (μg) | −0.446 | 6.26 | | 2.21 |
| Vitamin E (mg) | −0.419 | 8.73 | | 1.49 |
| Zn (mg) | −0.313 | 9.84 | | 2.19 |

Abbreviation：DII: Dietary Inflammatory Index
